# COVID-Related Perceptions of the Future and Well-Being Among Older Canadian Women

**DOI:** 10.1093/geroni/igab046.1971

**Published:** 2021-12-17

**Authors:** Hua (Poppy) Huo, Lauren Hytman, Cara Ryan, Nicky Newton

**Affiliations:** 1 Wilfrid Laurier University, Waterloo, Ontario, Canada; 2 Wilfried Laurier University, Waterloo, Ontario, Canada

## Abstract

Socioemotional Selectivity Theory (SST; Carstensen, 1993) posits that time horizons - or Future Time Perspective (FTP) - change with age and/or the priming of endings. Fung and Carstensen (2006) found that SARS-CoV in 2003 naturalistically primed fragility, with consequences for both FTP and well-being. The current SARS-CoV-2 (COVID-19) pandemic provides a similar context: During the early months of COVID-19, age and time horizon were related to greater emotional well-being for American adults (Carstensen et al., 2020); Dozois (2020) found that, for Canadian adults, anxiety and depression rose. The current study examines relationships between FTP, COVID-19 impact, and psychological well-being in older Canadian women (N = 190; Mage = 70.38). We found that COVID-19 impact and FTP were both related to well-being; additionally, COVID-19 impact moderated the relationship between FTP and well-being. The complexity of what remains or becomes increasingly important for older women during a global health crisis is discussed.

